# A rare femoral tumor in a young patient

**DOI:** 10.11604/pamj.2020.37.135.22667

**Published:** 2020-10-07

**Authors:** Faten Limaiem

**Affiliations:** 1 University of Tunis El Manar, Tunis Faculty of Medicine, 1007, Tunisia

**Keywords:** Parosteal osteosarcoma, bone, tumor, pathology

## Image in medicine

Parosteal osteosarcoma is a low-grade, bone-forming neoplasm that arises on the surface of bone. It accounts for about 4% of all osteosarcomas. An 18-year-old male patient with no particular past medical history, consulted for a painless mass in the right thigh that had appeared at the age of 17 years and progressively increased in volume. The physical examination revealed a 6 cm mass at its largest above the right popliteal fossa with knee flexion slightly limited. The X-ray revealed a well-limited mass in the lower third of the femur that was dense and attached to the metaphyseal cortex by a wide base. Histological examination of the biopsy specimen established the diagnosis of parosteal osteosarcoma. The patient underwent wide resection of the femoral tumor (A) preceded by a course of first-line chemotherapy. Histological examination showed a malignant mesenchymal proliferation, moderately cellular, made up of long, linear and eosinophilic material, sometimes calcified with no osteoblastic cells in the periphery (B). The tumor cells were spindle-shaped, with little eosinophilic cytoplasm and a long or ovoid, hyperchromatic, and moderately atypical nucleus. Mitoses were rare. There were no areas of dedifferentiation. Postoperative course was uneventful. During the one-year follow-up period, there was no recurrence or metastasis of the tumor. Parosteal osteosarcoma is characterized by its insidious growth and favorable prognosis. It rarely leads to metastasis. Its treatment is mainly surgical.

**Figure 1:**
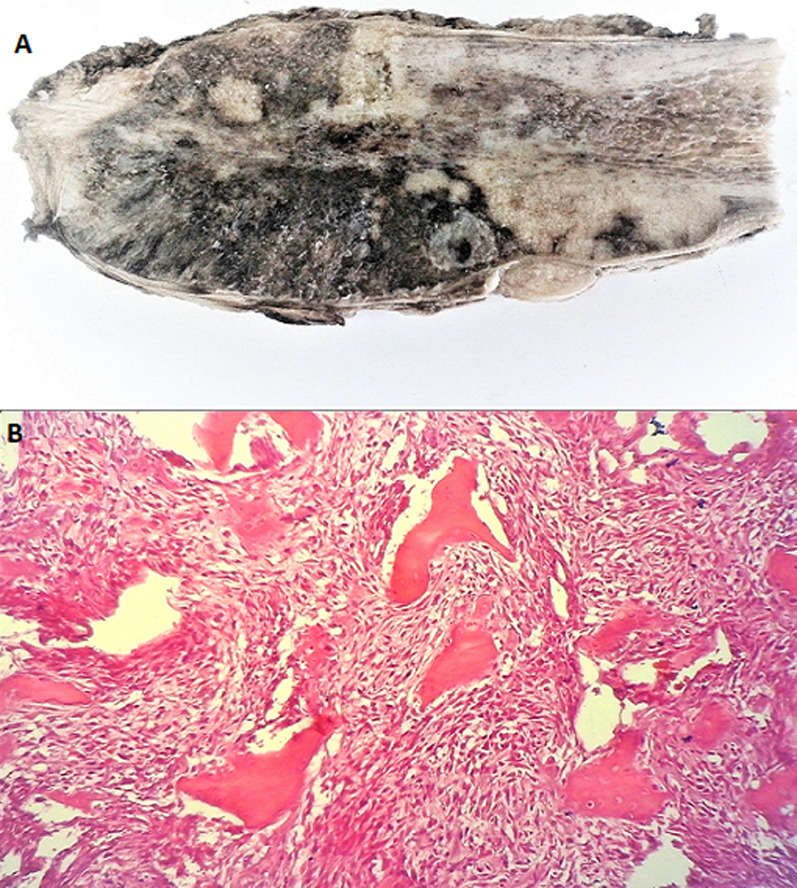
(A) macroscopic examination of the surgical specimen showing a lobulated, whitish tumor with focal cartilaginous zones measuring 6.3 cm x 2.8 cm, attached to the bone by a wide base; (B) photomicrograph of parosteal osteosarcoma showing mature-appearing bone without osteoblastic rimming, surrounded by a hypercellular fibroblastic stroma with moderate cytologic atypia, magnification (×200)

